# Substrate utilization and durability during prolonged intermittent exercise in elite road cyclists

**DOI:** 10.1007/s00421-024-05437-y

**Published:** 2024-03-05

**Authors:** Niels Ørtenblad, Magnus Zachariassen, Joachim Nielsen, Kasper Degn Gejl

**Affiliations:** Department of Sports Science and Clinical Biomechanics, University of Southern Denmark, Campusvej 55, 5230 Odense M, Denmark

**Keywords:** Endurance, Fatigue, Performance, Fat oxidation, Oxygen uptake, Carbohydrate oxidation

## Abstract

**Purpose:**

This study investigated the effects of prolonged intermittent cycling exercise on peak power output (PPO) and 6-min time-trial (6 min-TT) performance in elite and professional road cyclists. Moreover, the study aimed to determine whether changes in performance in the fatigued state could be predicted from substrate utilization during exercise and laboratory measures obtained in a fresh state.

**Methods:**

Twelve cyclists (age: 23 years [21;25]; body mass: 71.5 kg [66.7;76.8]; height: 181 cm [178;185]; $\dot{V}$O_2_peak: 73.6 ml kg^−1^ min^−1^ [71.2;76.0]) completed a graded submaximal cycling test to determine lactate threshold (LT_1_), gross efficiency (GE), and maximal fat oxidation (MFO) as well as power output during a maximal 6 min-TT (MPO_6 min_) in a fresh condition. On a separate day, the cyclists completed a 4-h intermittent cycling protocol with a high CHO intake (100 g h^−1^). Substrate utilization and PPO was measured hourly during the protocol, which was followed by another 6 min-TT.

**Results:**

MPO_6 min_ and PPO was reduced by 10% [4;15] and 6% [0;6], respectively, after the cycling protocol. These reductions were accompanied by reductions in the anaerobic energy contribution and $\dot{V}$O_2_peak, whereas the average $\dot{V}$O_2_ during the 6 min-TT was unchanged. Correlation analyses showed no strong associations between reductions in MPO_6 min_ and PPO and laboratory measures (i.e., LT_1_, GE, MFO, $\dot{V}$O_2_peak) obtained in the fresh condition. Additionally, fat oxidation rates during the cycling protocol were not related to changes in neither PPO nor MPO_6 min_.

**Conclusion:**

PPO and MPO_6 min_ were reduced following prolonged intermittent cycling, but the magnitude of these reductions could not be predicted from laboratory measures obtained in the fresh condition.

## Introduction

Decisive moments in road cycling races typically occur towards the final part of the race following prolonged fatiguing cycling. Consequently, the ability to generate high power output for short durations (e.g., breakaway or sprinting towards the finish line) after enduring hours of accumulated and intermittent work is of paramount importance for cycling performance (Padilla et al. 2001; Menaspà et al. 2015). Recent field studies have demonstrated negative effects of accumulated work on the maximal power output at given durations and the term “durability” has been coined to describe the ability to mitigate deteriorations in this relationship (Mateo-March et al. 2022; Muriel et al. 2022; Van Erp et al. 2021; Spragg et al. 2022, 2023). In this regard, successful road cyclists have been shown to exhibit smaller declines in maximal power output for given durations compared to less successful riders, following fixed amounts of accumulated work in the field (e.g., 45–50 kj∙kg^−1^) (Mateo-March et al. 2022; Van Erp et al. 2021).

Although the physiological determinants of durability in athletes are not yet clear, it is reasonable to hypothesize that it is multifactorial and that it involves the ability to withstand proposed mechanisms of fatigue during prolonged exercise (e.g., depletion of muscle and liver glycogen, reductions in exercise efficiency, impaired neuromuscular function etc.) (Ament and Verkerke 2009). Muscle glycogen and blood glucose are essential energy substrates during high-intensity exercise and ensuring the availability of carbohydrate (CHO) throughout endurance sport competitions is crucial for performance (Coyle et al. 1986; Jensen et al. 2020; Gejl et al. 2014). Therefore, continuous CHO supplementation is recommended during prolonged exercise at moderate-to-high intensities to maintain stable blood sugar levels and CHO oxidation (Stellingwerff and Cox 2014; Thomas et al. 2016). Currently elite endurance athletes generally consume higher amounts of CHO during competitions (i.e., 90–120 g h^−1^ of multiple transportable CHO) compared to previous practices (Podlogar et al. 2022; Podlogar and Wallis 2022).﻿ Another way to conserve CHO sources during prolonged exercise would be a shift towards a greater reliance on fat as a fuel source, since the utilization of CHO is inversely related to the oxidation of fatty acids. Consequently, possessing the ability to effectively utilize fat as an energy source alongside a high CHO intake could enhance durability during prolonged intermittent exercise. However, little is known about the extent to which fatty acids are oxidized during prolonged intermittent exercise (i.e., > 3 h) in elite endurance athletes under conditions of high CHO intake and how this is associated to durability.

In elite cycling, physiological evaluations are typically carried out in the laboratory under non-fatigued conditions, with $\dot{V}$O_2_max, lactate threshold (LT_1_), and gross efficiency (GE) being associated with endurance performance. However, it is uncertain to what extent such measurements can be employed to predict durability and performance in a fatigued state. A recent study investigated the relationship between traditional physiological laboratory based assessments (e.g., substrate utilization, $\dot{V}$O_2_max, LT, and GE) in a non-fatigued state and the relative reduction in exercise capacity following prolonged intermittent exercise (Spragg et al. 2023). In elite U23 cyclists, strong associations were observed between CHO oxidation, $\dot{V}$O_2_max, ventilatory threshold (VT_1_) and GE and relative deteriorations in critical power (CP) following 140 min of intermittent field cycling. However, not all studies have been able to demonstrate associations between laboratory measurements and durability (Valenzuela et al. 2023; Passfield and Doust 2000). Thus, in male professional cyclists, no significant associations were found between the decay in mean power output during a 20-min time trial following 4 h of cycling, and VT_1_, peak power output (PPO), and $\dot{V}$O_2_max(Valenzuela et al. 2023). Moreover, a study in less trained cyclists found no significant correlations between changes in 5-min maximal power output following 60 min of cycling at 60% $\dot{V}$O_2_peak and $\dot{V}$O_2_peak, MPO, LT measured in the fresh condition (Passfield and Doust 2000). The extent to which durability can be predicted from traditional laboratory tests remains uncertain and there might be a need to specifically assess durability in cyclists.

Through an intermittent protocol that simulates a road cycling race, the present study in professional and elite cyclists investigated the effects of 4 h of intermittent cycling exercise with a standardized high CHO intake on sprint performance [i.e., peak power output (PPO)] and performance during a 6-min time-trial (6 min-TT). Moreover, we examined substrate utilization during exercise and the extent to which this and traditional laboratory measures [i.e., LT, GE, $\dot{V}$O_2_max and maximal fat oxidation (MFO)] obtained in a fresh state can be used to predict changes in performance in the fatigued state. Based on the recent work by Spragg and colleagues (Spragg et al. 2022), we hypothesized that inter-individual variability in durability would be partly explained by the substrate utilization during exercise and the above-mentioned laboratory metrics, traditionally employed to describe endurance performance.

## Methods

### Participants

Twelve elite male road cyclists were recruited for the study (age, 23 years [21;25]; body mass, 71.5 kg [66.7;76.8]; height, 181 cm [178;185]; $\dot{V}$O_2_peak, 73.6 ml kg^−1^ min^−1^ [71.2;76.0]; LT_1_, 4.2 W kg^−1^ [3.9;4.5]). The cohort consisted of cyclists representing a UCI continental team (*n* = 7) and cyclists competing in national and international races on the elite level (*n* = 5). The participants were fully informed of any potential risk associated with the experiments before obtaining their consents and the experiments were conducted in accordance with the standards of the Declaration of Helsinki. The present study utilized procedures used during the ordinary training of the cyclists. As such, the present protocol did not require ethical approval according to the local ethical committee.

### Experimental overview

**Fig. 1:**
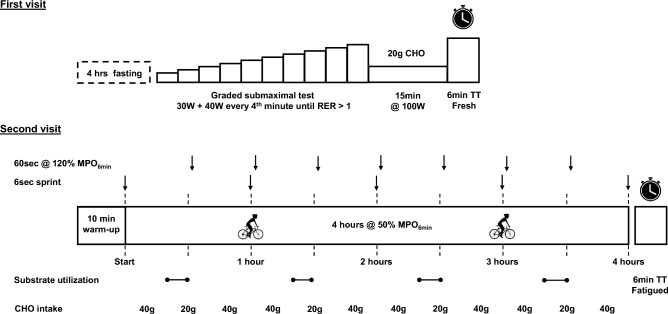
Schematic overview of the preliminary tests and the prolonged intermittent cycling protocol. The preliminary tests were preceded by 4 h of fasting and included a graded cycling test until the RER exceeded 1.00, followed by 15 min of cycling at 100 W including ingestion of 20 g CHO and a 6 min maximal time-trial (fresh condition). The prolonged cycling protocol was performed on the second laboratory visit and was commenced with a 10-min warm-up followed by 4 h of intermittent ergometer cycling and finally a 6-min maximal time-trial (fatigued condition). During most of the protocol, the intensity was 50% of the mean power output during the 6 min maximal time-trial conducted in the fresh condition (MPO_6 min_). In addition, every 30th minute, cyclists performed a 60 s interval at 120% of MPO_6 min_, while peak power output (PPO) was examined during 6 s of sprinting after the warm-up, after 60 min of cycling, and subsequently at hourly intervals. Throughout the cycling protocol, cyclists consumed 100 g CHO h^−1^, with 80 g CHO h^−1^ provided through beverages and an additional 20 g CHO h^−1^ from energy gels. Substrate utilization was determined at 50% MPO_6 min_ every hour by indirect calorimetry

The study was conducted in the final phase of the preparation period preceding the competitive part of the season (i.e., March and April). The cyclists visited the laboratory twice in the morning, separated by three to seven days (Fig. 1). The first visit involved preliminary tests including a submaximal step-test to obtain measurements of substrate utilization and gross efficiency, followed by a maximal 6 min-TT (i.e., fresh condition). The second visit (test day) consisted of a 4-h intermittent cycling protocol with a high CHO intake and repeated measurements of substrate utilization and peak power output (Fig. 1). Durability was examined through repeated 6 s peak power test during and after the 4 h of cycling as well as changes in maximal 6 min-TT conducted after the cycling protocol (i.e., fatigued state).

### Tests in the fresh condition (first visit)

The participants arrived at the laboratory after fasting for four hours and after refraining from high intensity exercise for 24 h. First, height and body weight were obtained and subsequently, a graded submaximal test on a cycling ergometer was initiated [Schoberer Rad Messtechnik (SRM), 117 GmbH Julich, Germany]. The test involved a 10-min period of rest, followed by 4-min steps starting at 30W, and with an increase of 40W every third minute until the RER value remained above 1.00 for a full minute. $\dot{V}$O_2_ and $\dot{V}$CO_2_ were measured throughout the test and used to calculate substrate utilization and GE at each workload (see details below). During the final 30 s of each workload, capillary blood was drawn from a fingertip to determine blood lactate concetration.

After completing the graded submaximal test, the participants received an energy gel containing 20 g of CHO (OTE, Leeds, UK) and continued cycling for 15 min at 100W. Then a maximal 6 min-TT (i.e., fresh condition) was conducted at a self-selected cadence and with verbal encouragement throughout the test. Participants were instructed to pace themselves to achieve the highest possible average power output. $\dot{V}$O_2_ and heart rate (HR) were recorded during the test. During each test, $\dot{V}$O_2_peak (see analytical procedures) and average power output (MPO_6 min_) was measured. HRpeak was defined as the highest measure of HR in each of the two MPO_6 min_ tests. The SRM ergometer was calibrated before each test.

### Prolonged intermittent cycling protocol and test in fatigued condition (second visit)

The prolonged intermittent cycling protocol was performed on the same SRM ergometer as described above, with calibration performed before each test. The cyclists refrained from high intensity exercise during the 24 h preceding the visits. The participants initiated the protocol with a 10-min warm-up, which included 5 min at 25% followed by 5 min at 50% of MPO_6 min_. The 4-h protocol was then started and comprised cycling at 50% of MPO_6 min_ interspersed with intervals of 1 min at 120% of MPO_6 min_ every 30 min (seven all in all) and maximal 6-s peak power tests every 60 min (five in total) (Fig. 1). After completing the 4 h protocol, subjects cycled for two minutes at 50% of MPO_6 min_ before the maximal 6 min-TT was conducted (i.e., same as preliminary test day but in a fatigued condition). The protocol was carried out at a self-selected cadence, except for the 6-s sprints, which were performed at a cadence of 100 RPM. HR was monitored throughout the protocol.

Except for the first sprint, all 6-s sprints were performed after 28 min of cycling at 50% of MPO_6 min_. From each of the 6-s sprints, the highest 3-s running average was defined as PPO. Before each sprint, a 3-s countdown was initiated, and verbal encouragement was provided throughout the sprint. Participants were instructed to exert their maximal effort during the entire sprint while remaining seated. Every second 1-min interval was performed after 30–32 min of cycling at 50% of MPO_6 min_, while the remaining 1-min intervals were performed after 30 min at 50% of MPO_6 min_, a 6-s sprint and an additional 2 min at 50% of MPO_6 min_ (Fig. 1). Substrate utilization was measured for 10 min at 50% of MPO_6 min_ every hour, starting after 20 min, and then every hour thereafter (see below). Participants were weighed before and after the entire protocol to estimate the degree of dehydration.

### Nutrition

Prior to the prolonged cycling protocol, participants were instructed to consume a CHO-enriched diet and to treat the nutritional preparation as they would before a real competition. Throughout the protocol, the participants were supplied with 100 g of CHO per hour, achieved by consuming an energy gel containing 20 g of CHO (OTE, Leeds, UK) and a 500 mL beverage containing 80 g of CHO (OTE, Leeds, UK) every hour (Fig. 1). The gels were ingested after every second 1-min interval (i.e., every hour), while consumption of the beverage was distributed across each 60-min period to prevent gastrointestinal issues (Fig. 1). In addition, the cyclists were allowed to consume water as desired. Importantly, all participants were accustomed to consume high amounts of carbohydrates during training and competition and none of the participants followed a “low carbohydrate − high fat” or other specific dietary strategies.

### Analytical procedures

#### $\dot{V}$O_*2*_* and *$\dot{V}$CO_*2*_

Using a mixing chamber system (Innocor, Innovision, Odense, Denmark) and based on the pulmonary ventilation and expiratory CO_2_ and O_2_ concentrations, both $\dot{V}$O_2_ and $\dot{V}$CO_2_ values were continuously sampled during both 6 min-TT, the graded submaximal test and repeatedly during the 4-h cycling protocol. Prior to each measurement, the gas analyzers were calibrated with two standard mixtures of gases containing 20.91% and 14.96% of O_2_ and 0.00% and 4.97% of CO_2_, respectively. The flow-meter was calibrated manually with a 3L syringe. The $\dot{V}$O_2_ and $\dot{V}$CO_2_ of each workload in the graded test were defined as the mean of measurements during the last minute of that workload. The highest mean 30-s values for $\dot{V}$O_2_ during the 6 min-TTs were identified as $\dot{V}$O_2_peak in the fresh and fatigued state, respectively.

### Calculation of substrate utilization, gross efficiency, and lactate threshold

During each step of the graded submaximal test and during the 4-h cycling protocol, the rates of fat oxidation and CHO oxidation were calculated using the stoichiometric equations developed by Jeukendrup and Wallis: fat oxidation rate = (1.695 × $\dot{V}$O_2_) − (1.701 × $\dot{V}$CO_2_); CHO oxidation rate = (4.210 × $\dot{V}$CO_2_) − (2.962 × $\dot{V}$O_2_), under the assumption that urinary nitrogen excretion was negligible (Jeukendrup and Wallis 2005). Maximal fat oxidation (MFO) was defined as the highest 1-min measurement recorded during the graded submaximal test. Unfortunately, due to a leaky valve, substrate utilization data were excluded for one participant. The first lactate threshold (LT_1_) was defined as the workload that resulted in the blood lactate concentration being 1 mmol L^−1^ blood higher than at rest. The workload corresponding to LT_1_ was identified by the workload-lactate relationship, by establishing a straight line through the lactate concentrations surrounding LT_1_.

Gross efficiency was calculated as the ratio between the external load (i.e., power output) and the total energy expenditure (EE), calculated from $\dot{V}$O_2_ and $\dot{V}$CO_2_. For the calculation of total EE, the following equation was used: EE = (0.550 × $\dot{V}$CO_2_) + (4.471 × $\dot{V}$O_2_) (Jeukendrup and Wallis 2005).

### Calculation of anaerobic and aerobic energy contribution during 6 min-TT

Average $\dot{V}$O_2_ in the last 60 s of each of the 9–10 submaximal steps (i.e., after attaining steady state $\dot{V}$O_2_) of the graded submaximal test were used to establish the individual power-$\dot{V}$O_2_ relationships. The anaerobic energy contribution was calculated based on the principle of determination of the accumulated oxygen deficit as described by Medbø et al. (1988). In brief, power output during the 6 min-TT’s in fresh and fatigued conditions was extracted in 10 s intervals and based on the power-$\dot{V}$O_2_ relationship the corresponding $\dot{V}$O_2_-demand was calculated. The oxygen deficit in each 10 s interval was then calculated as the difference between the calculated O_2_ demand and the measured O_2_ uptake and summarized for the whole 6-min exercise period.

### Statistical analyses

One-way ANOVA tests with repeated measures were used to investigate changes during the 4-h intermittent cycling protocol and a paired Students t-test was employed to compare results from the two maximal 6 min-TTs. The strengths of relationships between variables were assessed by Pearson´s correlation coefficient and [confidence intervals (CI)]. Although power-to-weight ratio offers a fair comparison of cycling performance between individuals, some correlation analyses were performed using absolute power output to avoid inter-dependency between the dependent and independent variables by normalizing both variables to body mass. Results are presented as mean and 95% [CI]. *P* < 0.05 were considered significant, while *P* = 0.05–0.10 were considered as tendencies. All statistical analyses and figures were produced using GraphPad Prism 6.07 (GraphPad Software, LLC, San Diego, CA 92108 USA).

## Results

### Exercise intensity and changes in heart rate during prolonged intermittent cycling

The average power output was 208 W [197;218] (2.9 W kg^−1^ [2.7;3.1]) during the steady portion of the cycling protocol (50% MPO_6 min_) and 498 W [472;523] (7.0 W kg^−1^ [6.6;7.4]) during the 1-min intervals (120% MPO_6 min_). On average, the intensity at 50% MPO_6 min_ corresponded to 70% of LT_1_ [68;72]. Since $\dot{V}$O_2_peak declined during the cycling protocol (see results later), the relative workload at 50% MPO_6 min_ increased from 54% [53;56] of pre $\dot{V}$O_2_peak to 59% [57;62] of post $\dot{V}$O_2_peak from the first to the fourth hour of exercise (mean effect: + 5% [2:8], *P* = 0.004).

HR increased from an average of 136 beats min^−1^ [130;142] (71% [69;73] of Pre HRpeak) during the first hour of exercise to 146 beats min^−1^ [138;153] (79% [75;82] of Post HRpeak) in the 4th hour of exercise (mean effect: + 7.5%-point [5:10], *P* < 0.0001) (2nd hour: 138 beats min^−1^ [131;144]; 3rd hour: 143 beats min^−1^ [135;150]). Body weight was slightly reduced following the cycling protocol (71.8 kg [66.7;76;8] to 71.3 kg [66.4;76.2], mean effect: − 0.4 kg [− 0.1; − 0.7], *P* = 0.003).

### Changes in substrate utilization and gross efficiency during prolonged intermittent cycling

**Fig. 2:**
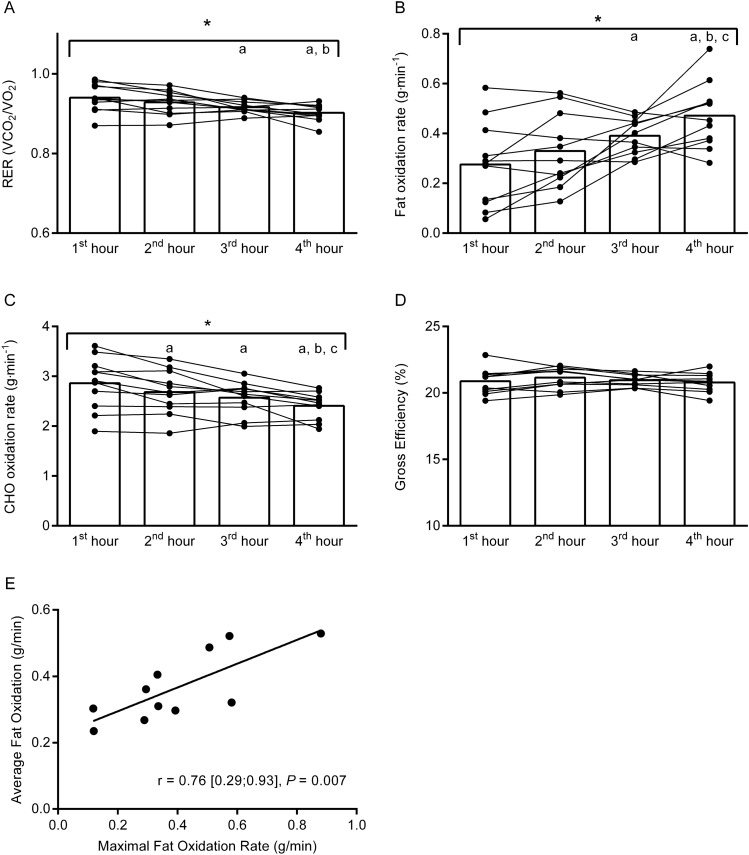
Effects of prolonged intermittent cycling exercise on the respiratory exchange ratio (RER) (**A**), the fat oxidation rate (**B**), the carbohydrate (CHO) oxidation rate (**C**), and gross efficiency (**D**). These outcomes were calculated based on measurements from indirect calorimetry performed from the 20th to the 30th min every hour of the prolonged cycling protocol. Individual data is indicated by black dots and connected by black lines. In addition, the relation between MFO after 4 h of fasting in the fresh state and the average FatOx rate during the cycling protocol is illustrated (E). *Main effect of time, *P* < 0.05, ^a^different from first hour, *P* < 0.05, ^b^different from second hour, *P* < 0.05, ^c^different from third hour, *P* < 0.05. Please see text for exact *P* values

The substrate utilization changed during the 4 h of intermittent cycling, as indicated by a decline in the RER (1st hour: 0.94 [0.92;0.96]; 2nd hour: 0.93 [0.91;0.95]: 3rd hour: 0.92 [0.91;0.93]; 4th hour: 0.90 [0.89;0.92], overall time-effect: *P* = 0.01) (Fig. 2A). A post hoc analysis revealed that the RER was significantly reduced during the 3rd and 4th hours of exercise compared to the 1st hour (mean effect 1st to 4th hour: − 0.04 [− 0.01; − 0.06], *P* = 0.01). Accordingly, the FatOx rate increased during the cycling protocol (1st hour: 0.28 g min^−1^ [0.16;0.39]; 2nd hour: 0.33 g min^−1^ [0.23;0.43]; 3rd hour: 0.39 g min^−1^ [0.34;0.44]; 4th hour: 0.47 g min^−1^ [0.38;0.56], overall time effect: *P* = 0.01). In addition, a post hoc analysis revealed that the FatOx rate was significantly elevated during the 3rd and 4th hour of exercise in comparison to the 1st hour (mean effect 1st to 4th hour: + 0.20 g min^−1^ [0.05;0.34], *P* = 0.01) (Fig. 2B). In line with this, the CHO oxidation was gradually reduced during the cycling protocol (1st hour: 2.9 g min^−1^ [2.5;3.2]; 2nd hour: 2.7 g min^−1^ [2.4;3.0]; 3rd hour: 2.6 g min^−1^ [2.4;2.8]; 4th hour: 2.4 g min^−1^ [2.2;2.6], overall time-effect: *P* = 0.002) (mean effect 1st to 4th hour: − 0.45 g min^−1^ [− 0,19: − 0.72], *P* = 0.003) (Fig. 2C).

There was no significant change in GE during the cycling protocol, (1st to 4th hour: 20.9% [20.2;21.5] to 20.8% [20.3;21.3], mean effect: 0.0%-point [0.0;0.0], *P* = 0.75 (Fig. 2D) or $\dot{V}$O_2_ (1st to 4th hour: 2.85 L O_2_ min^−1^ [2.68;3.01] to 2.88 L O_2_ min^−1^ [2.70;3.06], mean effect: 0.03 L O_2_ min^−1^ [− 0.05:0.11], *P* = 0.43).

Notably, there was a strong correlation (*r* = 0.76 [0.29;0.93], *P* = 0.007) between MFO in the fresh condition and the average FatOx during the prolonged cycling protocol, indicating that interindividual differences in fat oxidative capabilities were maintained despite a high CHO intake during the prolonged cycling protocol (Fig. 2E).

### Changes in maximal cycling performance by prolonged intermittent cycling.

**Fig. 3:**
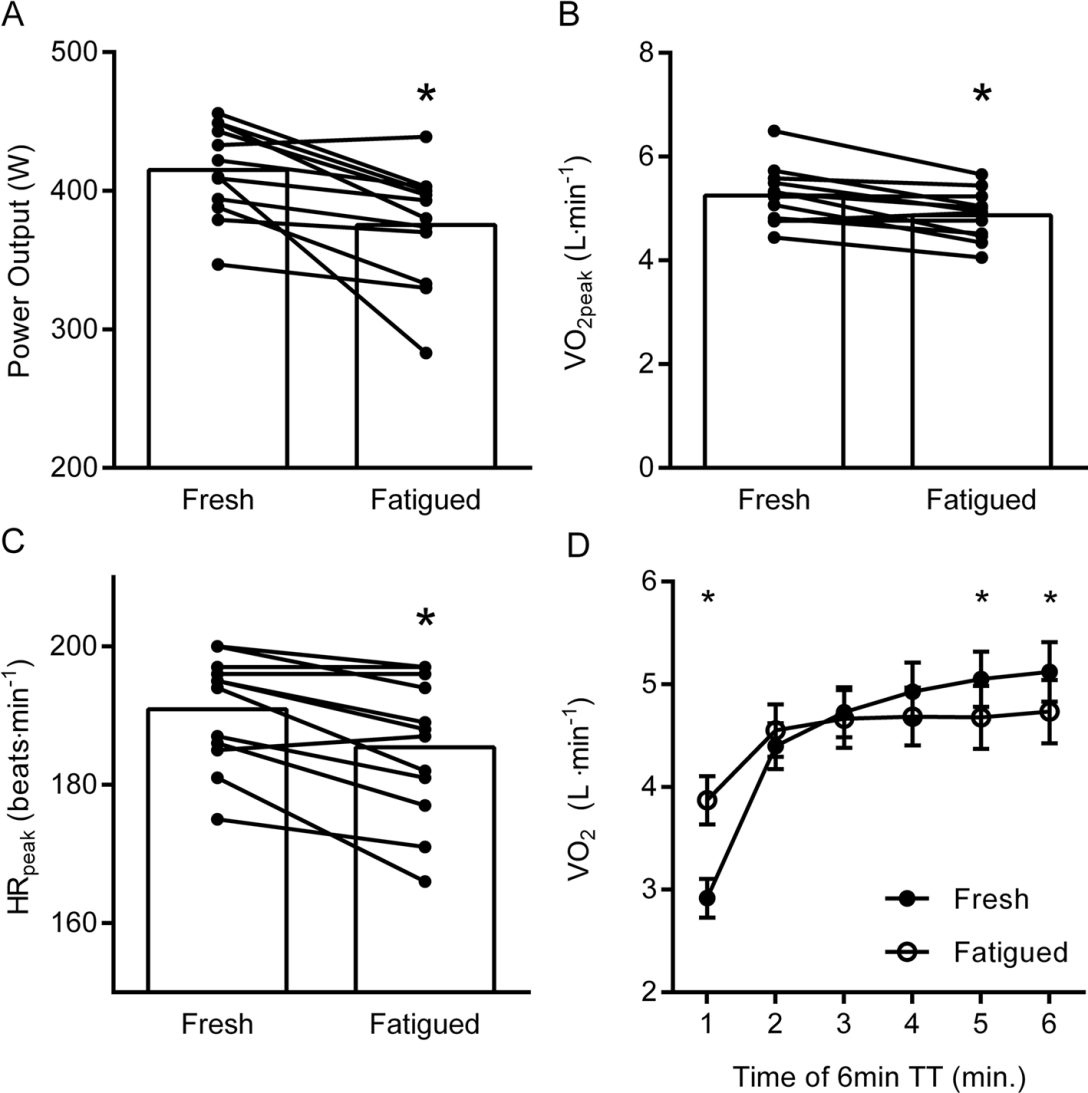
Mean power output (MPO_6 min_) (**A**), $\dot{V}$O_2_peak (**B**), HRpeak (**C**), and average $\dot{V}$O_2_ for each minute during the 6 min maximal time-trial in the fresh condition (Fresh) and following prolonged intermittent cycling exercise (Fatigued) (**D**). In panel **A**–**C**, individual data is indicated by black dots and connected by black lines. Panel **D** shows mean and 95% CI. *Different from fresh condition, *P* < 0.05. Please see text for exact *P* values

MPO_6 min_ was significantly reduced from 415 W [395;436] to 375 W [349;402] following the prolonged intermittent cycling protocol (mean effect: − 40 W [− 17; − 62], *P* = 0.003) (Fig. 3A). Furthermore, during the MPO_6 min_-test in fatigued compared to fresh condition there was a 7% [3;11] decrease in $\dot{V}$O_2_peak (5.25 L O_2_ min^−1^ [4.90;5.60] to 4.87 L O_2_ min^−1^ [4.57;5.16], mean effect: − 0.38 L O_2_ min^−1^ [− 0.16: − 0.61], *P* = 0.003) and a 3% [1;6] decrease in HRpeak (191 beats min^−1^ [186;196] to 185 beats min^−1^ [179;192], mean effect: − 6 beats min^−1^ [− 2: − 9], *P* = 0.003) (Fig. 3B, C). While the average $\dot{V}$O_2_ during the 6-min test was not different between fresh and fatigued conditions (4.51 L O_2_ min^−1^ [4.27;4.75] vs. 4.53 L O_2_ min^−1^ [4.26;4.80], mean effect: 0.02 L O_2_ min^−1^ [− 0.17:0.21], *P* = 0.88) (Fig. 3D) there were clear differences during the tests. Thus, when divided into 1-min intervals, a divergence in $\dot{V}$O_2_kinetics appeared between conditions (Fig. 3D). Thus, $\dot{V}$O_2_ was higher during the first minute in the fatigued state (2.92 L O_2_ min^−1^ [2.73;3.11] vs. 3.87 L O_2_ min^−1^ [3.64;4.11], mean effect: + 0.95 L O_2_ min^−1^ [0.78:1.12], *P* < 0.001), but lower during the 5th (5.05 L O_2_ min^−1^ [4.78;5.32] vs. 4.68 L O_2_ min^−1^ [4.37;4.99], mean effect: − 0.37 L O_2_ min^−1^ [− 0.11: − 0.63], *P* = 0.01) and 6th minute (5.12 L O_2_ min^−1^ vs. 4.73 L O_2_ min^−1^ [4.43;5.04], mean effect: − 0.4 L O_2_ min^−1^ [− 0.1: − 0.6], *P* = 0.006).

Calculations of the anaerobic energy contribution showed a reduction from 8.6% [7.5;9.7] in the fresh condition to 3.0% [2.3;3.8] (*P* < 0.0001) in the fatigued condition, meaning that the aerobic energy contribution increased from 91.4% [90.3;92.5] to 97.0% [96.7;97.2] (*P* < 0.0001). In absolute terms, the accumulated O_2_-deficit during the 6 min-TT was reduced from 2.8 L min^−1^ [2.3;3.3] to 1.0 L min^−1^ [0.6;1.3] (*P* < 0.0001) after the cycling protocol.

**Fig. 4:**
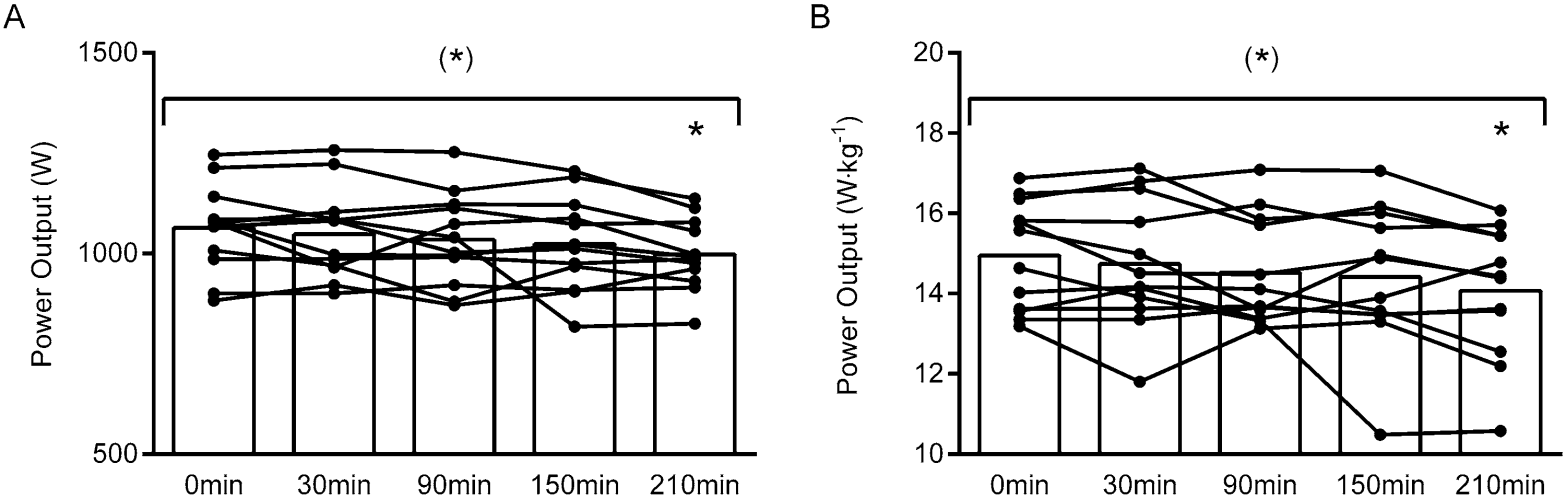
Effects of prolonged intermittent cycling exercise on absolute peak power output (PPO) (**A**) and PPO normalized to body mass (**B**). Individual data are indicated by black dots and connected by black lines. *Different from 0 min, *P* < 0.05, (*)Tendency towards an overall time effect (*P* = 0.05–0.10). Please see text for exact *P* values

Regarding PPO, there was a tendency towards an overall reduction during the 4 h of cycling (Fresh: 1064 W [994;1134]; 1st hour: 1048 W [977;1119]; 2nd hour: 1035 W [962;1108]; 3rd hour: 1024 W [949;1099]; 4th hour: 998 W [949;1099], overall time effect: *P* = 0.09). A post hos analysis revealed a significant reduction in PPO after 4 h of cycling compared to pre-exercise (mean effect: − 67 W [− 4; − 129], *P* = 0.04) (Fig. 4A, B).

### *Relationship between substrate utilization and ΔMPO*_*6 min*_* and ΔPPO*

**Fig. 5:**
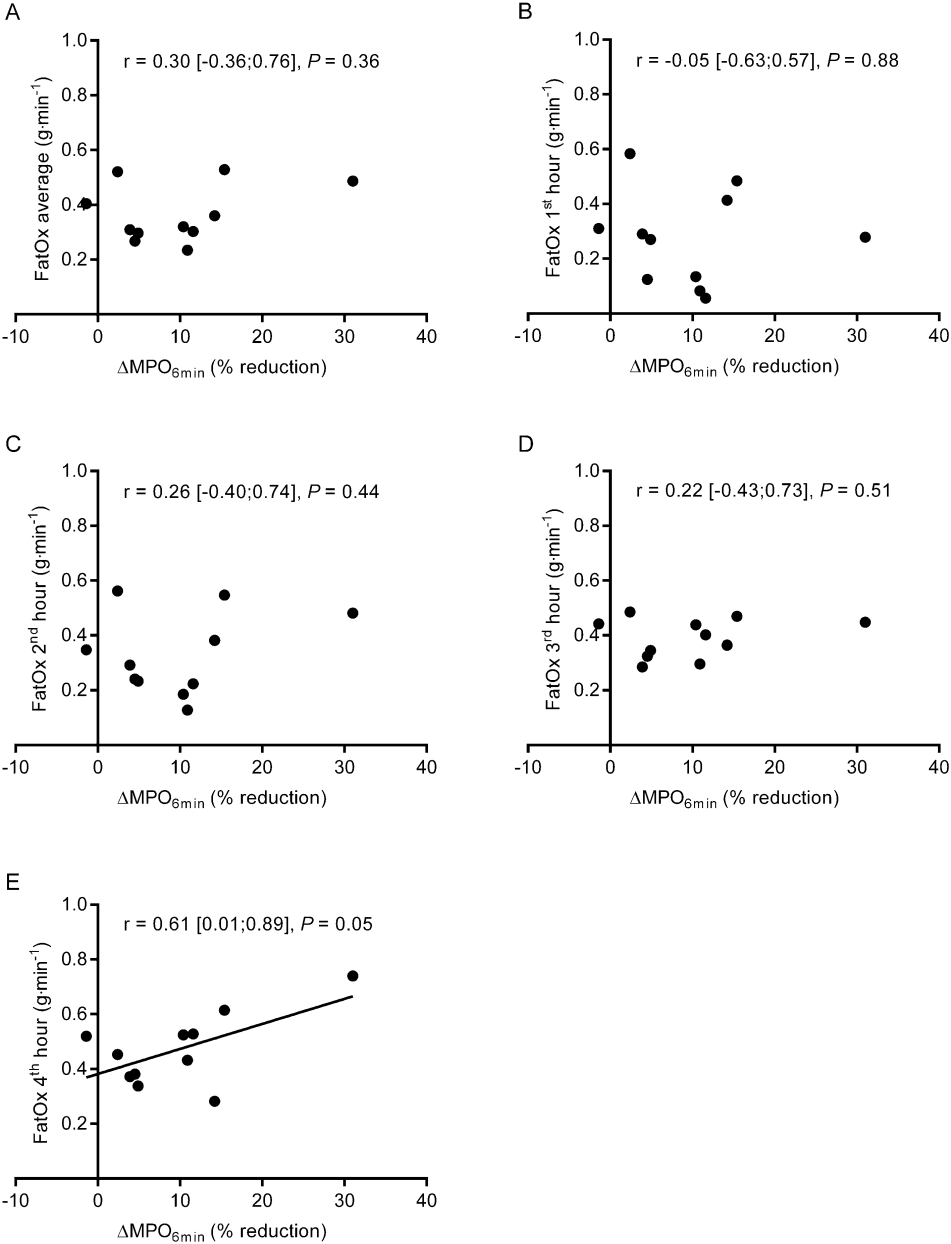
Relationships between relative reductions in mean power output during the 6 min maximal time-trial following the prolonged intermittent cycling protocol (ΔMPO_6 min_) and (**A**) the average fat oxidation rate during the entire cycling protocol, as well as the fat oxidation rates during the first (**B**), second (**C**), third (**D**) and fourth (**E**) hour of the protocol. Pearson´s correlation coefficient, 95% CI, and *P* values from correlation analyses are shown in each panel

We next investigated if the substrate utilization correlated with changes in MPO_6 min_ and PPO during the cycling protocol. The average (1st to 4th hour) or the time-specific FatOx rate at 1st, 2nd or 3rd hour did not show any strong correlations with ΔMPO_6 min_ and the data are most compatible with poor correlations (Fig. 5A–D). However, during the 4th hour a fair correlation was found with a confidence interval ranging from a poor to a good correlation (Fig. 5E), showing that higher FatOx rates were associated with larger declines in performance in the last portion of the cycling protocol (i.e., after 3 h of exercise).

The CHO oxidation during the cycling protocol was not associated with ΔMPO_6 min_ (average: *r* = 0.03, [− 0.58:0.62], *P* = 0.92; 1st hour: *r* = 0.17 [− 0.48:0.70], *P* = 0.62; 2nd hour: *r* = 0.04 [− 0.57:0.63], *P* = 0.90; 3rd hour: *r* = 0.12 CI [− 0.52:0.67], *P* = 0.73; 4th hour: *r* = − 0.36 [− 0.79:0.31], *P* = 0.28). Additionally, ΔPPO was not related to neither the average CHO oxidation during exercise nor the CHO oxidation during the first three hours of cycling (average: *r* = − 0.12 [− 0.57:0.62], *P* = 0.73; 1st hour: *r* = 0.11 [− 0.53:0.66], *P* = 0.75; 2nd hour: *r* = − 0.05 [− 0.63:0.57], *P* = 0.89; 3rd hour: *r* = − 0.14 [− 0.68:0.50], *P* = 0.68. However, CHO oxidation rates were negatively associated with ΔPPO during the 4th hour (i.e., high CHO oxidation associated with low reduction in PPO) (*r* = − 0.59 [− 0.88:0.00], *P* = 0.05).

### Relationship between laboratory measures in fresh condition and ΔMPO_6 min_ and ΔPPO

Under the present conditions, correlation analyses unveiled that neither of the laboratory-based outcomes obtained in a fresh condition (i.e., MPO_6 min_, $\dot{V}$O_2max_, LT_1_, MFO, and GE_230 W_) were correlated with ΔMPO_6 min_ or ΔPPO (Tables 1, 2).

**Table 1:** Correlations between relative changes in MPO_6 min_ and laboratory measures

	MPO_6 min_ (W)	MPO_6 min_ (W kg^−^^1^)	*V*O_2_peak (ml kg^−^^1^∙min^−^^1^)	LT_1_ (W kg^−^^1^)	LT_1_ (% MPO_6 min_)	MFO (g min^−^^1^)	GE_230 W_ (%)
Pearson’s r	0.19	− 0.23	− 0.05	− 0.27	− 0.19	0.20	0.00
95% CI	[− 43:69]	[− 71:40]	[− 61:54]	[− 73:36]	[− 69:43]	[− 43:69]	[− 57:57]
*P* value	0.56	0.48	0.88	0.40	0.55	0.54	1.00

*MPO* mean power output during maximal 6 min test, *VO*_*2*_*peak* peak oxygen uptake during maximal 6 min test, *LT*_*1*_ first lactate threshold, *MFO* maximal fat oxidation, *GE*_*230 W*_ gross efficiency at 230 W during the graded submaximal test

**Table 2:** Correlations between relative changes in PPO and laboratory measures

	MPO_6 min_ (W)	MPO_6 min_ (W kg^-1^)	*V*O_2_peak (ml kg^−^^1^ min^−^^1^)	LT_1_ (W kg^−^^1^)	LT_1_ (% MPO_6 min_)	MFO (g min^−^^1^)	GE_230 W_ (%)
Pearson’s r	0.02	− 0.48	− 0.37	− 0.50	− 0.36	0.41	− 0.19
95% CI	[− 56:59]	[− 83:13]	[− 78:26]	[− 84:10]	[− 79:30]	[− 22:80]	[− 69:43]
*P* value	0.94	0.12	0.24	0.10	0.27	0.19	0.56

*MPO* mean power output during maximal 6 min test, *VO*_*2*_*peak* peak oxygen uptake during maximal 6 min test, *LT*_*1*_ first lactate threshold, *MFO* maximal fat oxidation, *GE*_*230 W*_ gross efficiency at 230 W during the graded submaximal test

### Relationship between laboratory measures and performance following fatiguing exercise

**Fig. 6:**
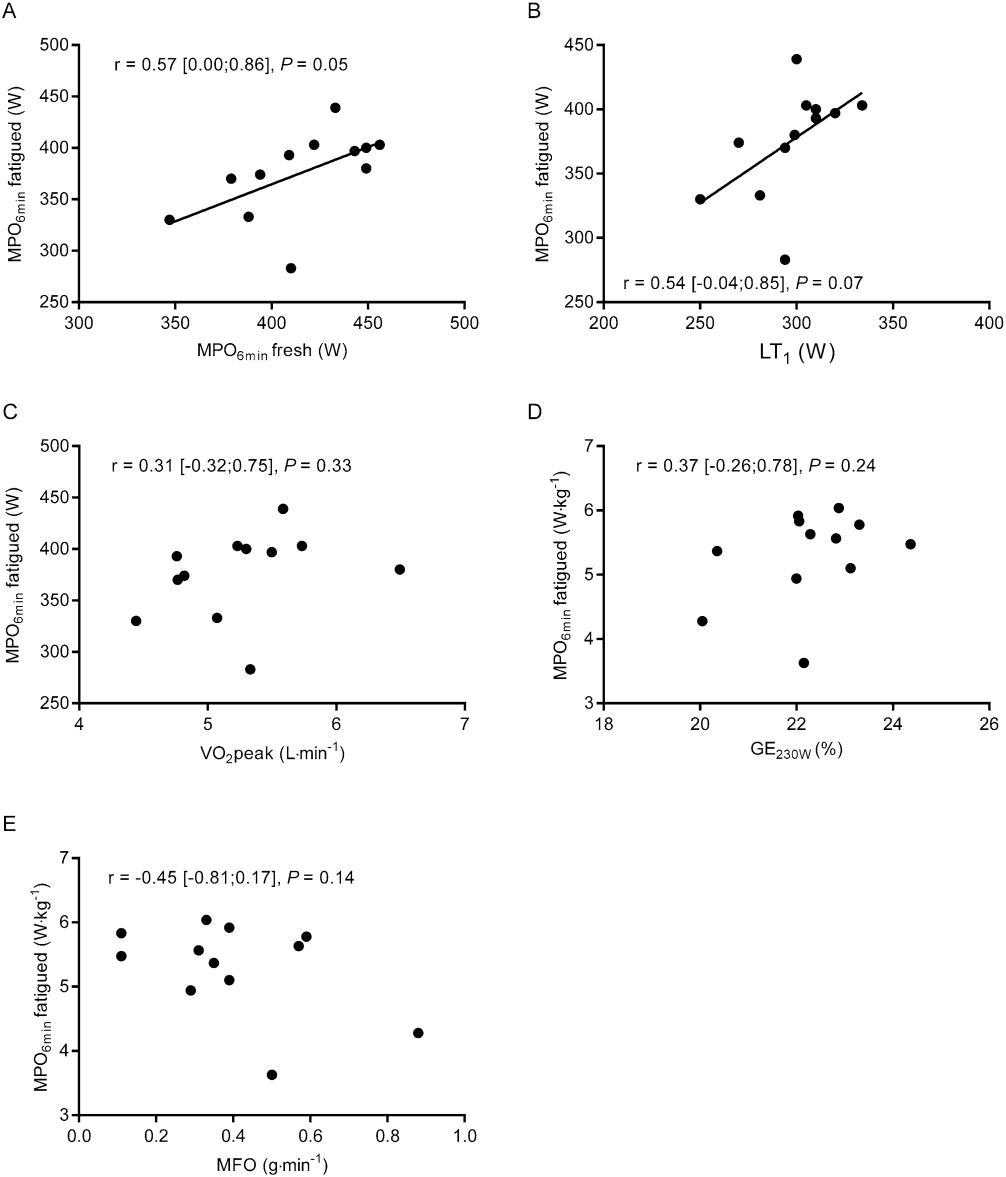
Relationships between absolute mean power output during the 6 min maximal time-trial (MPO_6 min_) performed following prolonged intermittent ergometer cycling and laboratory-based measures obtained in the fresh condition: MPO_6 min_ (**A**), power output at lactate threshold (**B**), $\dot{V}$O_2_peak (**C**), gross efficiency at 230 W (**D**), and the maximal fat oxidation rate (**E**). Pearson´s correlation coefficient, 95% CI, and *P* values from correlation analyses are shown in each panel

Besides the ability to withstand relative reductions in performance, the ranking in road cycling races is largely influenced by the absolute exercise capacity (i.e., W or W kg^−1^) following prolonged accumulated intermittent exercise. Correlation analyses unveiled associations tending to statistical significance between MPO_6 min_ in the fatigued condition and MPO_6 min_ (*r* = 0.57 [0.00;0.86], *P* = 0.05) and LT_1_ (*r* = 0.54 [− 0.04;0.85], *P* = 0.07) in the fresh condition (Fig. 6A, B), while no significant correlations were observed between MPO_6 min_ in the fatigued state and $\dot{V}$O_2_peak (*r* = 0.31 [− 0.32;0.75], *P* = 0.33) and GE at 230 W (*r* = 0.37 [− 0.26;0.78], *P* = 0.24) in the fresh state (Fig. 6C, D). Finally, no significant correlation was observed between MFO measured in the fresh condition and performance during MPO_6 min_ in the fatigued state (*r* = − 0.45 [− 0.81;0.17], *P* = 0.14) (Fig. 6E).

## Discussion

In the present cohort of elite and professional road cyclists, 4 h of intermittent cycling exercise with provision of 100 g CHO h^−1^ compromised both sprint performance (PPO) and maximal aerobic exercise performance (MPO_6 min_). The cyclists demonstrated a gradual increase in the FatOx rate during the 4 h of intermittent exercise despite ingesting large amounts of CHO. Moreover, under the present conditions, we could not demonstrate strong associations between durability (i.e., relative changes in MPO_6 min_ and PPO) and laboratory-based endurance measures obtained in the fresh condition or the FatOx rate during exercise.

### Performance following prolonged intermittent exercise

Here, we designed a controlled prolonged intermittent cycling protocol simulating some of the demands of road cycling races, and by this protocol fatigue was induced, as demonstrated by 9% and 6% impairments in a 6-min TT and PPO, respectively. These reductions align with findings from previous studies involving elite cyclists subjected to prolonged exercise (Spragg et al. 2023; Valenzuela et al. 2023; Gejl et al. 2014). Notably, $\dot{V}$O_2_peak was also reduced in the fatigued condition (Fig. 3D), and as expected this reduction displayed a strong correlation with the concurrent reduction in MPO_6 min_ (*r* = 0.79, *P* = 0.003) (Fig. 3D). Moreover, the reduction in $\dot{V}$O_2_peak was accompanied by a reduction in HRpeak of 6 ± 5 bpm indicating that the reductions in $\dot{V}$O_2_peak and MPO_6 min_ could, at least partly, be ascribed to a reduction in O_2_ delivery. Unfortunately, the mechanisms underlying the reductions in $\dot{V}$O_2_peak and HRpeak cannot be elucidated from the present study. While $\dot{V}$O_2_peak was reduced, the average $\dot{V}$O_2_ during the 6 min time-trials showed no alteration after the cycling protocol. Thus, given the decrease in average power, the reduction in MPO_6 min_ was likely explained by the observed reduction in the anaerobic energy contribution from 9.6 to 3.0% (change in accumulated O_2_-deficit: 2.8 L min^−1^ to 1.0 L min^−1^). Another measure of performance at high exercise intensities is *W´* (i.e., the amount of work that can be done above the CP), and reductions in *W*´ have previously been observed following prolonged intermittent exercise in both professional cyclists (Spragg et al. 2022) and recreationally active individuals (Bitel et al. 2023). Unfortunately, a robust measure of *W´* could not be determined from the test used in the present study (i.e., 6 s PPO and 6 min-TT). Taken together, prolonged intermittent exercise apparently compromise the anaerobic capacity and W´, and eventually performance in elite cyclists.

The decline in $\dot{V}$O_2_peak during exercise implied that the relative metabolic load at 50% MPO_6 min_ was increased from 54 to 59% of $\dot{V}$O_2_peak from the first to the fourth hour of exercise. Consequently, the relative exercise intensity was at a higher level within the moderate intensity domain and for coaches and athletes such changes are likely important to consider to acquire a valid estimate of the training and competition load. In addition, prior research has demonstrated a drift in $\dot{V}$O_2_ (i.e., $\dot{V}$O_2_ slow component) during prolonged submaximal exercise, likely attributed to changes in substrate utilization and muscle fiber recruitment patterns, resulting in a loss of efficiency (Burke et al. 2017; Xu and Montgomery 1995; Passfield and Doust 2000; Hagberg et al. 1978; Pringle et al. 2003). In this regard, a recent study in endurance athletes demonstrated that prolonged moderate intensity exercise reduced the power output at the boundary between the moderate and heavy intensity domain due to reductions in GE and metabolic energy expenditure at this transition (Stevenson et al. 2022). Indeed, such drifts in $\dot{V}$O_2_ or GE have previously been shown to be diminished in endurance trained individuals (Casaburi et al. 1987; Russell et al. 2002; MacDougall et al. 2022), and present data is in line with this, showing no changes in $\dot{V}$O_2_ or GE despite a gradual shift towards a greater reliance on fat as an energy source.

### Fat oxidation and durability

The group of elite and professional cyclists exhibited a wide range of relative changes of MPO_6 min_ following the cycling protocol (− 31% to + 1%) and we aimed to investigate the relation between these changes and changes in substrate utilization during the cycling protocol, as well as MFO in the fresh condition. Considering the crucial role of muscle glycogen in sustaining high-intensity exercise performance (Gollnick et al. 1974; Balsom et al. 1999; Rockwell et al. 2003), the FatOx rate may have a substantial impact on the preservation of muscle glycogen. As muscle- and liver glycogen was not estimated directly in the present study, the utilization of glycogen can therefore only be estimated. Based on the average CHO oxidation of 2.63 ± 0.37 g min^−1^ at 50% of MPO_6 min_, and for simplicity neglecting the increased CHO oxidation during the high intensity intervals, the cumulative CHO oxidation was ~ 630 g during the cycling protocol. Assuming that 90 g of the ingested CHO was oxidized per hour (Podlogar et al. 2022), then approximately 270 g of CHO was derived from liver and muscle glycogen. As previously seen in less trained individuals (Fell et al. 2021), these estimations indicate that despite a high CHO intake there is a substantial use of muscle glycogen in active muscles of elite cyclists during this type of exercise, and consequently mobilization and oxidation of other fuel sources may eventually becomes important. Nonetheless, when employing correlation analysis, we could not demonstrate strong associations between measures of fat oxidation during exercise and durability in the present scenario. Moreover, MFO in the fresh and fasted condition (i.e., 4 h of fasting) did not display a correlation with durability.

As illustrated in Fig. 2B, the FatOx rate was relatively low for most cyclists during the first two hours of cycling (i.e., ≤ 0.4 g min^−1^ and < 30% of total energy expenditure in 9 riders), meaning that the potential preservation of muscle glycogen during this phase was limited. In addition, a comparison of the cyclist with the highest average FatOx rate (0.53 g min^−1^) and the one with the lowest average FatOx rate (0.23 g min^−1^), and identical $\dot{V}$O_2_ at 50% of MPO_6 min_ (~ 3.1 L min^−1^), revealed a total difference in CHO oxidation of ~ 110 g and a difference of 18 g in the fourth hour of cycling (avg. total CHO oxidation: 632 g [573;691]. Therefore, it is possible that modest inter-individual variations in the FatOx rates may have prevented the detection of associations between FatOx rates and durability.

Importantly, in the current study, achieving statistical significance in the correlation analyses required an *r* value of 0.58 or higher, given the sample size. Consequently, only variables explaining 36% or more of the variance in durability were considered statistically significant. It is, therefore, worth noting that based on the confidence intervals, it is plausible that pertinent associations may not have been detected. The recruitment of elite and professional cyclists involved challenges in terms of sample size for the present study and studies with larger cohorts of cyclists of this caliber are needed to gain further insight into the significance of FatOx capacity for durability.

### Laboratory-based measurements and durability

Only a limited number of studies have investigated predictors of durability in trained endurance athletes and our findings align with previous research failing to establish associations between declines in time-trial performance after prolonged cycling exercise and laboratory-based metrics measured under fresh conditions (e.g., $\dot{V}$O_2_max, VT_1_ and VT_2_) (Valenzuela et al. 2023; Passfield and Doust 2000). Also, in a recent study of professional cyclists (Spragg et al. 2023) no association was found between FatOx capacity at 200 W and 300 W in a fresh condition and durability (*r* = 0.18, 95% CI [− 51:73] and *r* = − 0.08, 95% CI [− 68:58], respectively), which is consistent with findings of the present study (Table 1). However, they identified robust correlations between durability (i.e., relative reductions in CP) and laboratory-based measures obtained in a fresh condition (e.g., $\dot{V}$O_2_max, VT_1_, VT_2_ and GE), which were not evident in the present study. In this regard, it should be noted that confidence intervals were wide in both studies, partly due to relatively low sample sizes, and there was a large overlap between confidence intervals of the correlations between durability and laboratory-based measures. Consequently, part of the observed differences between studies were probably due to uncertain estimates, emphasizing the need for larger sample sizes to obtain more precise estimates regarding the predictability of durability from laboratory-based metrics measured under fresh conditions.

Since the participants in the study by Spragg and colleagues were somewhat comparable to those in the present study (i.e., elite cyclists with similar $\dot{V}$O_2_max), the contradictory findings could also be due to differences in methodological approaches, i.e., different duration and intensity of the fatiguing cycling protocols as well as differences in the CHO intake during exercise. We aimed to use a realistic protocol in terms of the duration of road cycling races, while the protocol used by Spragg and colleagues were somewhat shorter (i.e., 4 h vs. 2 h 12 min). On the other hand, the volume of high-intensity cycling was much higher in the latter study. Such differences in fatiguing protocols likely affect the contribution from mechanisms underlying fatigue and durability (e.g., glycogen utilization, fiber contractile properties, and central factors) (Jensen et al. 2020; Hvid et al. 2013; Millet and Lepers 2004), and thereby also laboratory-based predictors of durability. Furthermore, the determinants of durability may vary depending on the consumption of CHO during exercise. While it is generally accepted that CHO intake during prolonged exercise prevents reductions in performance by preserving liver glycogen and blood glucose levels and perhaps also muscle glycogen, the optimal dose of CHO is debated (Podlogar and Wallis 2022). Current recommendations do not advise a CHO intake above 90–100 g h^−1^ since it seems that higher doses do not enhance endurance performance further (Thomas et al. 2016; Stellingwerff and Cox 2014). In the present study we adhered to these recommendations by providing 100 g CHO h^−1^. Importantly, such a high CHO intake suppresses the FatOx rate in endurance athletes (Gejl et al. 2018; Fell et al. 2021), which may minimize the importance of a high FatOx capacity for performance and durability as long as sufficient glucose sources are present. Nonetheless, there was a significant correlation between MFO in the fasted state and the FatOx rate during the first hour of the cycling protocol, indicating a carry-over effect despite the high CHO intake. However, under the current conditions this was not positively associated with durability. The extent to which the FatOx capacity determines durability under conditions where the CHO availability may become limited should be further examined in the future. In this regard, we suggest that future studies in elite endurance athletes examine the interaction between carbohydrate availability and the FatOx rate and its importance for durability under various conditions (i.e., different types of endurance exercise and fueling strategies).

Based on the present findings, and most other cross-sectional studies, durability seems unrelated to traditional laboratory measures of endurance performance. In line, a recent study in untrained individuals showed that training induced changes in $\dot{V}$O_2_max and power at LT_1_ following 10 weeks of training were not associated with changes in durability (Matomäki et al. 2023). Future research in elite endurance athletes should investigate changes in durability in response to different training strategies and the extent to which changes in durability can be explained by changes in traditional measures of endurance performance.

## Conclusions

In conclusion, both sprint performance (PPO) and maximal aerobic exercise performance (MPO_6 min_) were impaired following 4 h of intermittent exercise in male elite cyclists. These deteriorations were not strongly correlated with neither substrate utilization during exercise nor laboratory-based measures obtained in the fresh condition. Additionally, the elite cyclists exhibited a gradual increase in the FatOx rate during the prolonged cycling protocol with ingestion 100 g CHO h^−1^.

## Data Availability

Data is available from the corresponding author upon reasonable request.
