# Correction: Substrate utilization and durability during prolonged intermittent exercise in elite road cyclists

**DOI:** 10.1007/s00421-024-05479-2

**Published:** 2024-05-08

**Authors:** Niels Ørtenblad, Magnus Zachariassen, Joachim Nielsen, Kasper Degn Gejl

**Affiliations:** Department of Sports Science and Clinical Biomechanics, University of Southern Denmark, Campusvej 55, 5230 Odense M, Denmark

**Correction: European Journal of Applied Physiology** 10.1007/s00421-024-05437-y

In the original version of this article, the unit on the Y-axis of Fig. 3B was wrong. The Fig. 3 should have appeared as shown below.

**Fig. 3:**
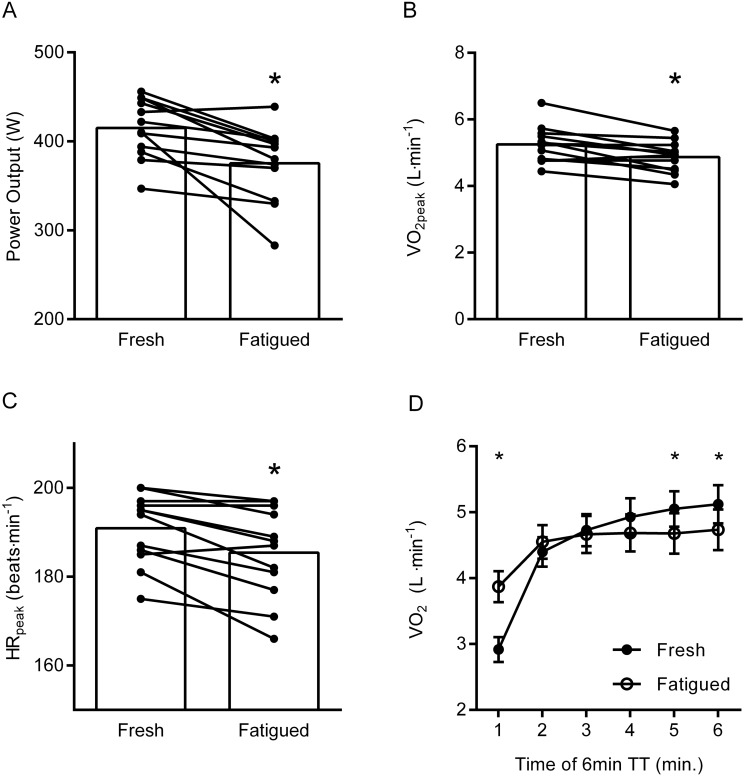
Mean power output (MPO_6 min_) (**A**), $\dot{V}$O_2_peak (**B**), HRpeak (**C**), and average $\dot{V}$O_2_ for each minute during the 6 min maximal time-trial in the fresh condition (Fresh) and following prolonged intermittent cycling exercise (Fatigued) (**D**). In panel **A**–**C**, individual data is indicated by black dots and connected by black lines. Panel **D** shows mean and 95% CI. *Different from fresh condition, *P* < 0.05. Please see text for exact *P* values

The original article has been corrected.

